# A rapid and simple MALDI-TOF MS lipid profiling method for differentiating *Mycobacterium ulcerans* from *Mycobacterium marinum*

**DOI:** 10.1128/jcm.01400-24

**Published:** 2025-01-27

**Authors:** Takeshi Komine, Hanako Fukano, Mitsunori Yoshida, Yuji Miyamoto, Makoto Nakaya, Azumi Fujinaga, Kohei Doke, Yoshihiko Hoshino

**Affiliations:** 1 Department of Mycobacteriology, Leprosy Research Center, National Institute of Infectious Diseases 231182 , Higashimurayama, Tokyo, Japan; 2 Application Department, Microbiology & Diagnostics MID Division, Bruker Japan K.K., Yokohama, Kanagawa, Japan; The University of North Carolina at Chapel Hill School of Medicine, Chapel Hill, North Carolina, USA

**Keywords:** lipid profiling, MALDI-TOF MS, nontuberculous mycobacteria, Buruli ulcer, *Mycobacterium ulcerans*, *Mycobacterium marinum*

## Abstract

*Mycobacterium ulcerans*, a slow-growing nontuberculous mycobacterium, causes Buruli ulcer, a neglected tropical disease. Distinguishing *M. ulcerans* from related species, including *Mycobacterium marinum*, poses challenges with respect to making accurate identifications. In this study, we developed a rapid and simple identification method based on mycobacterial lipid profiles and used matrix-assisted laser desorption/ionization time-of-flight mass spectrometry (MALDI-TOF MS) to analyze the lipid profiles of *M. ulcerans* (*n* = 35) and *M. marinum* (*n* = 19) isolates. Bacterial colonies pre-cultured on 2% Ogawa egg slants for 2 months were collected, and total lipids were extracted using an MBT Lipid Xtract kit. Spectra were obtained in negative ion mode using a MALDI Biotyper Sirius system, with ClinProTools v3.0 being used to analyze the spectra based on the application of three algorithms (genetic algorithm [GA], supervised neural network [SNN], and quick classifier [QC)]). Cross-validation was performed using a 20% leave-out set randomly selected from the samples. Models generated using GA, SNN, and QC showed cross-validation values of 100%, 100%, and 97.9%, respectively, and all algorithms achieved 100% recognition capability values. Our findings indicate that MALDI-TOF analysis of lipid profiles can accurately differentiate two mycobacterial species (*M. ulcerans* and *M. marinum*) that are difficult to distinguish using conventional protein-targeting methods.

**IMPORTANCE:**

Buruli ulcer, caused by *Mycobacterium ulcerans*, is a neglected tropical disease. However, distinguishing *M. ulcerans* from related species, including *Mycobacterium marinum*, presents certain challenges. In this study, we demonstrate the utility of a rapid yet simple method for differentiating isolates of these mycobacteria based on their lipid profiles using matrix-assisted laser desorption/ionization time-of-flight mass spectrometry. This new approach can accurately identify species that are otherwise difficult to distinguish using conventional techniques. This represents a significant diagnostic advance for clinical laboratories, in that it enables a more rapid and precise identification, thereby leading to earlier treatment initiation and more appropriate treatment regimens for infections caused by these bacteria.

## INTRODUCTION

*Mycobacterium ulcerans*, a slow-growing nontuberculous mycobacterium, is the etiological agent of Buruli ulcer, a severe intractable type of skin lesion (1, 2). Although this tends to be a largely neglected tropical disease, it is particularly prevalent in Africa and Australia, with 4.55 cases per 100,000 reported in Liberia between 2010 and 2017 and 5.5 cases per 100,000 in Victoria, Australia, in 2018 (3–5). Cases have also been reported in Japan, where a unique subspecies, *M. ulcerans* subsp. *shinshuense*, was isolated (6).

The delayed diagnosis and treatment of Buruli ulcers can lead to significant sequelae, making early detection and intervention essential for reducing complications (7). Consequently, a novel, rapid, and simple method for identifying *M. ulcerans* is required. However, current methods tend to be time-consuming and labor-intensive. Although sequencing of the 16S rRNA gene is commonly used to identify microbial pathogens, the sequence similarity between *M. ulcerans* and the related species *Mycobacterium marinum* is >98.65 (8), and hence additional phylogenetic analyses of several housekeeping genes and/or the detection of insertion sequences and specific genes (IS*2404*, IS*2606*, and *KR-B*) of *M. ulcerans* are required (9–12). However, IS*2404* is also present in *Mycobacterium pseudoshottsii* and some strains of *M. marinum*, whereas IS*2606* is found in *Mycobacterium lentiflavum* (10, 13). Accordingly, the combined use of these insertion sequences, along with the *KR-B* gene, is required for the specific detection of *M. ulcerans*. Even so, alone, these markers would not be sufficient for the accurate identification of this pathogen.

Matrix-assisted laser desorption/ionization time-of-flight mass spectrometry (MALDI-TOF MS) is a cost-effective, rapid, and accurate method for microbial identification based on an analysis of protein signatures (14, 15). In recent years, MALDI-TOF MS has played an important role in the identification of different species of *Mycobacterium* in clinical laboratories (16–18). For example, the MALDI Biotyper system manufactured by Bruker (Germany), incorporating the MBT Mycobacteria library v7, can be used to identify 182 of the 201 currently known mycobacterial species (https://www.bruker.com/en/products-and-solutions/microbiology-and-diagnostics/microbial-identification/maldi-biotyper-library-ruo.html, accessed 1/4/2024). However, current proteomic strategies using MALDI-TOF MS are unable to discriminate between species belonging to several mycobacterial groups, such as the *M. ulcerans*/*M. marinum* complex (15, 19). Indeed, a case has been reported in which a strain that matched *M. marinum* based on proteomic identification transpired to be *M. ulcerans* subsp. *shinshuense* (20).

Mycobacteria are characterized by a unique type of cell wall that is rich in proteins, polysaccharides, and lipids, and certain mycobacterial species are characterized by the presence of unique, highly antigenic virulence-associated lipids, referred to as glycopeptidolipids, located on the surface of the cell wall (21–23). In this regard, it has been established that species/subspecies of the *M. tuberculosis* complex can be identified based on lipidomic approaches (24), whereas subspecies of *Mycobacterium abscessus* can be identified using lipid profiling and machine learning (25). Notably, *M. ulcerans* produces mycolactone, a diffusible, cytotoxic, and immunosuppressive lipid-like toxin (26, 27), whereas apart from rare strains containing mycolactone F, *M. marinum* lacks lipid-like toxins (28). In this study, we exploited these differences in mycobacterial lipids for species and subspecies identification, for which we developed a simple and rapid method, in which MALDI-TOF MS was applied in conjunction with machine learning for the identification of *M. ulcerans* based on its total lipid profile.

## MATERIALS AND METHODS

### Mycobacterial isolates

A total of 54 clinical isolates (12 *M*. *ulcerans*, 23 *M*. *ulcerans* subsp. *shinshuense*, and 19 *M*. *marinum*) were used in this study (Table S1). *M. ulcerans* isolates collected from 1983 to 1999 were obtained from the Institute of Tropical Medicine in Australia, Benin, Ghana, the Ivory Coast, Malaysia, and Togo. *M. ulcerans* subsp. *shinshuense* and *M. marinum* were isolated in Japan between 2012 and 2021. *M. ulcerans* subsp. *shinshuense* isolates were classified based on 16S rRNA gene sequencing, according to Nakanaga et al. (29).

### Sample preparation and spectra acquisition using MALDI-TOF MS

Frozen stocks of the *Mycobacterium* isolates were revived on 2% Ogawa egg slants for 2 months. Total mycobacterial lipids were extracted from loopfuls of single colonies processed using an MBT Lipid Xtract kit (Bruker, Germany) according to the manufacturer’s instructions. Spectra of the lipid profiles were obtained using a MALDI Biotyper Sirius system (Bruker) in negative ion mode with mass-to-charge ratios (*m/z*) in the range of 750 to 4,000 using flexControl software v3.4. To determine whether mycolactone was detectable under these measurement conditions, we used synthetic mycolactone A/B, kindly provided by Dr. Yoshito Kishi of Harvard University, as a standard and performed measurements in the range from *m/z* 500 to 1,000.

### Predictive models and validation

The spectra were selected and sorted based on a range of statistical analyses, including the *t*-test, analysis of variance, and the Wilcoxon or Kruskal–Wallis test, in ClinProTools v3.0 (Bruker, Germany) with default settings, and were also analyzed using three algorithms, namely, the genetic algorithm (GA), supervised neural network (SNN), and quick classifier (QC), available in ClinProTools v3.0, to generate predictive models (30). The QC-based analysis in this study was performed using *P* values obtained from Wilcoxon/Kruskal–Wallis tests and a sort/weight mode. Cross-validation and recognition capability values, as criteria of the robustness of the generated models, were calculated and are expressed as a percentage of samples correctly assigned to the studied groups, as described below. We generated a model using 80% of spectra randomly selected and then applied this model to assess remaining 20% of spectra. This process was repeated 10 times, with the average of these values being taken as the cross-validation value. We also generated a model using all spectra, which were assessed with the model to establish how well the underlying data characteristics were captured (= recognition capability value). Furthermore, principal component analysis (PCA) using ClinProTools v3.0 was performed for dimension reduction of the data obtained for *M. ulcerans* and *M. marinum*.

## RESULTS

**Fig 1:**
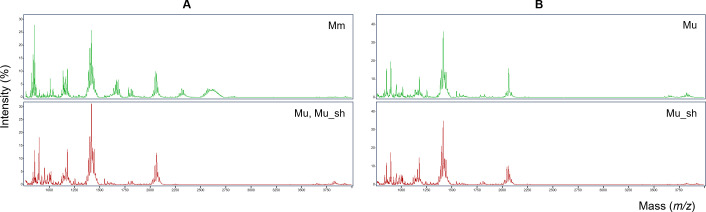
Average mass spectra of mycobacteria within the *Mycobacterium ulcerans/marinum* complex. (**A**) Comparison between *M. marinum* (green) and *M. ulcerans*, including *M. ulcerans* subsp. *shinshuense* (red). (**B**) Comparison between *M. ulcerans* (green) and *M. ulcerans* subsp. *shinshuense* (red). The lipid spectra were acquired in negative ion mode using a MALDI Biotyper Sirius system. The ellipses represent the standard deviation of the class average of the peak areas/intensities or the 95% confidence interval, which is the standard deviation weighted by the reciprocal number of data points. Mu, *M. ulcerans*; Mu_sh, *M. ulcerans* subsp. *shinshuense*; and Mm, *M. marinum*.

**Fig 2:**
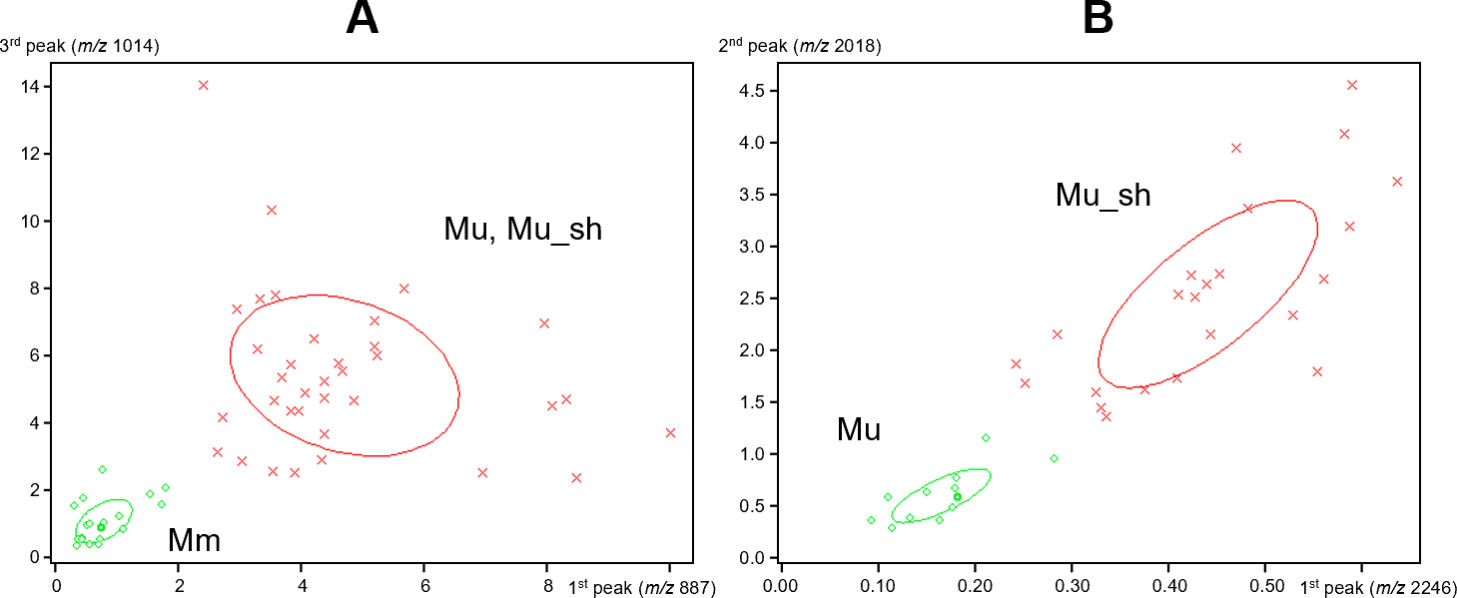
Strain distribution map. (**A**) Comparison between *Mycobacterium marinum* (green) and *M. ulcerans*, including *M. ulcerans* subsp. *shinshuense* (red). (**B**) Comparison between *M. ulcerans* (green) and *M. ulcerans* subsp. *shinshuense* (red). Mu, *M. ulcerans*; Mu_sh, *M. ulcerans* subsp. *shinshuense*; Mm, *M. marinum*. The *x*-axis shows the peak area or intensity values with respect to the most relevant peak for distinguishing the two groups (green and red). The *y*-axis shows the peak area or intensity values with respect to the second or third most relevant peak for distinguishing the two groups.

Total lipid spectra of the two targeted mycobacterial species were acquired in the negative ion mode using MALDI-TOF MS (Fig. 1). A strain distribution map based on the lipid profiles successfully distinguished *M. ulcerans* from *M. marinum* (Fig. 2A). In addition, *M. ulcerans* and *M. ulcerans* subsp. *shinshuense* were subgrouped (Fig. 2B).

In the comparison between *M. ulcerans* (including *M. ulcerans* subsp. *shinshuense*) and *M. marinum*, analyses using the GA, SNN, and QC classification algorithms revealed cross-validation values of 100%, 100%, and 97.9%, respectively. The peaks used for model cross-validation are shown in Table 1; Fig. S1. The 100% cross-validation values obtained using the GA and SNN models were based on five peaks (*m/z* 835.6, 1663.2, 2299.0, 2601.5, and 2616.5) and seven peaks (*m/z* 1651.7, 1678.6, 1693.7, 2284.0, 2326.2, 2340.2, and 2596.7), respectively. Using all three algorithms, in each case, we obtained recognition capability values of 100%. In the comparison between *M. ulcerans* (excluding strains isolated from Japan) and *M. ulcerans* subsp. *shinshuense*, analyses performed using the GA, SNN, and QC models yielded cross-validation values of 91.4%, 93.2%, and 92.7%, respectively, with the value obtained using the SNN model, generated based on peaks *m/z* 1613.5 and 2245.9, being marginally the highest (Table 1; Fig. S2). Unsupervised PCA further confirmed that *M. ulcerans* was distinguished from *M. marinum* and that *M. ulcerans* subsp. *shinshuense* was distinct from *M. ulcerans* (Fig. S3). Given that the analytical approach adopted in this study is based on lipid profiling performed using machine learning, it is not necessary to identify each peak. However, we did observe interesting differences between the profiles of *M. marinum* and *M. ulcerans*, with that of the former being characterized by signal areas mainly at *m/z* 1,663 and 2,312, which were not observed in the profiles of *M. ulcerans* (Fig. 1). The main signal plus *m/z* 16 and the main signal minus *m/z* 63 were detected in both areas, indicating that there may be structural similarities between the compounds in these areas. Using a mycolactone A/B standard, we also sought to establish whether mycolactone could be detected under the conditions imposed in this study. However, we were unable to mycolactone in either the negative mode or in the matrix of the MBT Lipid Xtract kit.

**TABLE 1:** Results of classifying models based on three algorithms^*a*^

Compared groups	Number of peaks	Model	Cross-validation value	Recognition capability value	Peaks used for the model (*m/z*)
*Mycobacterium ulcerans* and *M. ulcerans* subsp. *shinshuense* vs *M. marinum*	98	GA	100	100	835.6, 1663.2, 2299.0, 2601.5, 2616.5
		SNN	100	100	1651.7, 1678.6, 1693.7, 2284.0, 2326.2, 2340.2, 2596.7
		QC	97.9	100	885.5, 887.1, 1651.7
*M. ulcerans* vs *M. ulcerans* subsp. *shinshuense*	76	GA	91.4	100	1139.3, 1399.1, 1613.5, 2245.9, 3673.7
		SNN	93.2	100	1613.5, 2245.9
		QC	92.7	100	1161.2, 1399.0, 1613.5, 1808.3, 1991.3, 2018.4, 2045.9, 2245.9, 3385.4, 3673.7, 3687.3, 3849.8, 3922.3, 3936.2

^*a*^ GA, genetic algorithm; SNN, supervised neural network; QC, quick classifier.

## DISCUSSION

Early diagnosis and treatment are key measures that can contribute to minimizing the morbidity and costs associated with Buruli ulcers and can also prevent long-term disability (31). However, during the early stages of infection, distinguishing between the nodular lesions caused by *M. ulcerans* and *M. marinum* can prove challenging, with multiple time-consuming molecular biological examinations being required to reliably identify these two mycobacterial pathogens (9–12). In this study, we demonstrate that a lipidomics approach based on MALDI-TOF MS analysis can be used to effectively distinguish *M. ulcerans* from *M. marinum*. Our findings revealed that GA models based on five peaks or SNN models based on seven peaks would be suitable for distinguishing these two species. The high sensitivity and specificity of this method indicate that when operating in both positive and negative ion modes, which mainly target proteins and lipids, respectively, this approach could serve as a more powerful identification tool in clinical microbiology.

A lipidomic strategy has previously been applied to discriminate the *M. abscessus* complex (comprising subspecies *abscessus*, *bolletii*, and *massiliense*) based on differences in lipids and glycolipids, including glycosylated glycopeptidolipids (32). The clinical isolates of *M. ulcerans* detected in Japan comprise a genetically unique group, *M. ulcerans* subsp. *shinshuense*, which compared with the *M. ulcerans* sub-type from West Africa are more susceptible to streptomycin, kanamycin, and clarithromycin in *in vitro* susceptibility tests (29). Consequently, in the future, sub-typing *M. ulcerans* clinical isolates could contribute to determining antimicrobial susceptibilities and facilitate the acquisition of accurate epidemiological information. In this regard, our findings in the present study indicate that mycobacterial subspecies contain subspecies-specific lipids, and we thus believe that the strategy reported herein would be useful for sub-typing *M. ulcerans*. The two peaks used in the SNN model would be comparatively suitable for sub-typing *M. ulcerans*. Consequently, a future challenge will be to determine which lipid components are specific to each sub-type.

Although phenotyping based on traits such as pigment production is often used to characterize *M. ulcerans* and *M. marinum*, these traits can be unstable. *M. ulcerans*, *M. ulcerans* subsp. *shinshuense*, and *M. marinum* are generally characterized as being non-chromogenic, scotochromogenic, and photochromogenic, respectively (28, 29, 33). However, phenotypically atypical strains are occasionally isolated (29, 34, 35), and, moreover, the pigmentation of these mycobacteria can differ depending on the test temperature and the presence or absence of a mycolactone-producing giant plasmid (36, 37). Comparatively, the MALDI-TOF MS method described herein is less influenced by atypical phenotypes or the subjectivity and proficiency of assessors in performing phenotypic analyses and enables mechanically stable identification.

This study does, nevertheless, have certain limitations. Firstly, the number of isolates examined was relatively small, and the geographical distribution of these isolates and time spans over which they were isolated were also limited. Secondly, the method developed in this study is based on total lipid fingerprints, and it remains unclear as to which specific lipids contribute to species and subspecies diversification. Moreover, when using both the GA and SNN models, the peaks used for differentiation did not match the previously reported peaks of mycolactones (26, 37), which tends to indicate that these peaks correspond to differences in other lipids (such as cell wall lipids) unique to each species.

Despite these limitations, the MALDI-TOF MS method we developed has the distinct advantage of rapid analysis. Compared with conventional PCR methods, which from the extraction of cultured bacteria to identification can take up to several hours to complete, the newly developed method can be performed in approximately 20 min. Moreover, it requires the use of fewer reagents and is more cost-effective. We thus believe that this rapid yet simple lipidomic-based method will be useful for the rapid diagnosis of Buruli ulcers, thereby facilitating the early initiation of appropriate treatment regimens.

## SUPPLEMENTAL MATERIAL

Supplemental material jcm.01400-24-s0001.docx Figures S1 to S3; Table S1.

ASM does not own the copyrights to Supplemental Material that may be linked to, or accessed through, an article. The authors have granted ASM a non-exclusive, world-wide license to publish the Supplemental Material files. Please contact the corresponding author directly for reuse.
