# Supplementary material for: A rapid and simple MALDI-TOF MS lipid profiling method for differentiating Mycobacterium ulcerans from Mycobacterium marinum
Source: J Clin Microbiol. 2025 Jan 27;63(3):e01400-24. doi: 10.1128/jcm.01400-24 (PMC11898672; doi:10.1128/jcm.01400-24)
Supplement: Supplemental material — Figures S1 to S3; Table S1. [file jcm.01400-24-s0001.docx]

**SUPPLEMENTAL REFERENCES**

1. Nakanaga K, Hoshino Y, Yotsu RR, Makino M, Ishii N. 2011. Nineteen Cases of Buruli Ulcer Diagnosed in Japan from 1980 to 2010. *J Clin Microbiol* 49. https://doi.org/10.1128/jcm.00783-11.
2. Das S, Pettersson BMF, Behra PRK, Mallick A, Cheramie M, Ramesh M, Shirreff L, DuCote T, Dasgupta S, Ennis DG, Kirsebom LA. 2018. Extensive genomic diversity among *Mycobacterium marinum* strains revealed by whole genome sequencing. *Sci Rep* 8(1):12040. doi: 10.1038/s41598-018-30152-y. Erratum in: 2020 *Sci Rep* 10(1):5246. doi: 10.1038/s41598-020-61218-5. PMID: 30104693; PMCID: PMC6089878.
3. Phillips R, Horsfield C, Kuijper S, Lartey A, Tetteh I, Etuaful S, Nyamekye B, Awuah P, Nyarko KM, Osei-Sarpong F, Lucas S, Kolk AHJ, Wansbrough-Jones M. 2005. Sensitivity of PCR Targeting the IS2404 Insertion Sequence of *Mycobacterium ulcerans* in an Assay Using Punch Biopsy Specimens for Diagnosis of Buruli Ulcer. *J Clin Microbiol* 43:3650–3656. https://doi.org/10.1128/jcm.43.8.3650-3656.2005.

**SUPPLEMENTAL FIGURE LEGENDS**

**FIG S1** Example of analysis using the novel MALDI-TOF MS lipid profiling method showing a comparison of the average spectrum of *Mycobacterium marinum* (green line) and the average spectrum of *M. ulcerans*, including *M. ulcerans* subsp. *shinshuense* (red line). Ninety-eight peaks were detected in the spectra and were assessed using each of the three evaluated analytical models: GA, genetic algorithm (upper); SNN, supervised neural network (middle); and QC, quick classifier (lower). Blue areas show detected peaks and red areas show the peaks of each model identified as applicability domains that distinguish the two mycobacterial pathogens. To clarify the differences between the spectra of the two groups, each red area is shown as an enlarged spectrum below each model. The GA, SNN, and QC models identified five, seven, and three peaks, respectively.

**FIG S2** Example of analysis using the novel MALDI-TOF MS lipid profiling method showing a comparison of the average spectrum of *Mycobacterium ulcerans* (green line) and the average spectrum of *M. ulcerans* subsp. *shinshuense* (red line). Seventy-six peaks were detected in the spectra and were assessed using each of the three evaluated analytical models: GA, genetic algorithm (upper); SNN, supervised neural network (middle); and QC, quick classifier (lower). Blue areas show detected peaks and red areas show the peaks of each model identified as applicability domains that distinguish the two mycobacterial pathogens. To clarify the differences between the spectra of the two groups, each red area is shown as an enlarged spectrum below each model. The GA, SNN, and QC models identified five, two, and 14 peaks, respectively.

**FIG S3** Results of principal component analysis (PCA) of *Mycobacterium ulcerans* and *M. marinum*. Unsupervised analysis performed using ClinProTools v3.0 distinguishes the two species (A, *M. ulcerans* and *M. marinum*) and sub-species (B, *M. ulcerans* and *M. ulcerans* subsp. *shinshuense*) based on their lipid profiles. a, a 3D PCA plot for principal components (PCs) 1–3. b, explained variance ratio of PC1–10.

**FIG S1**

**
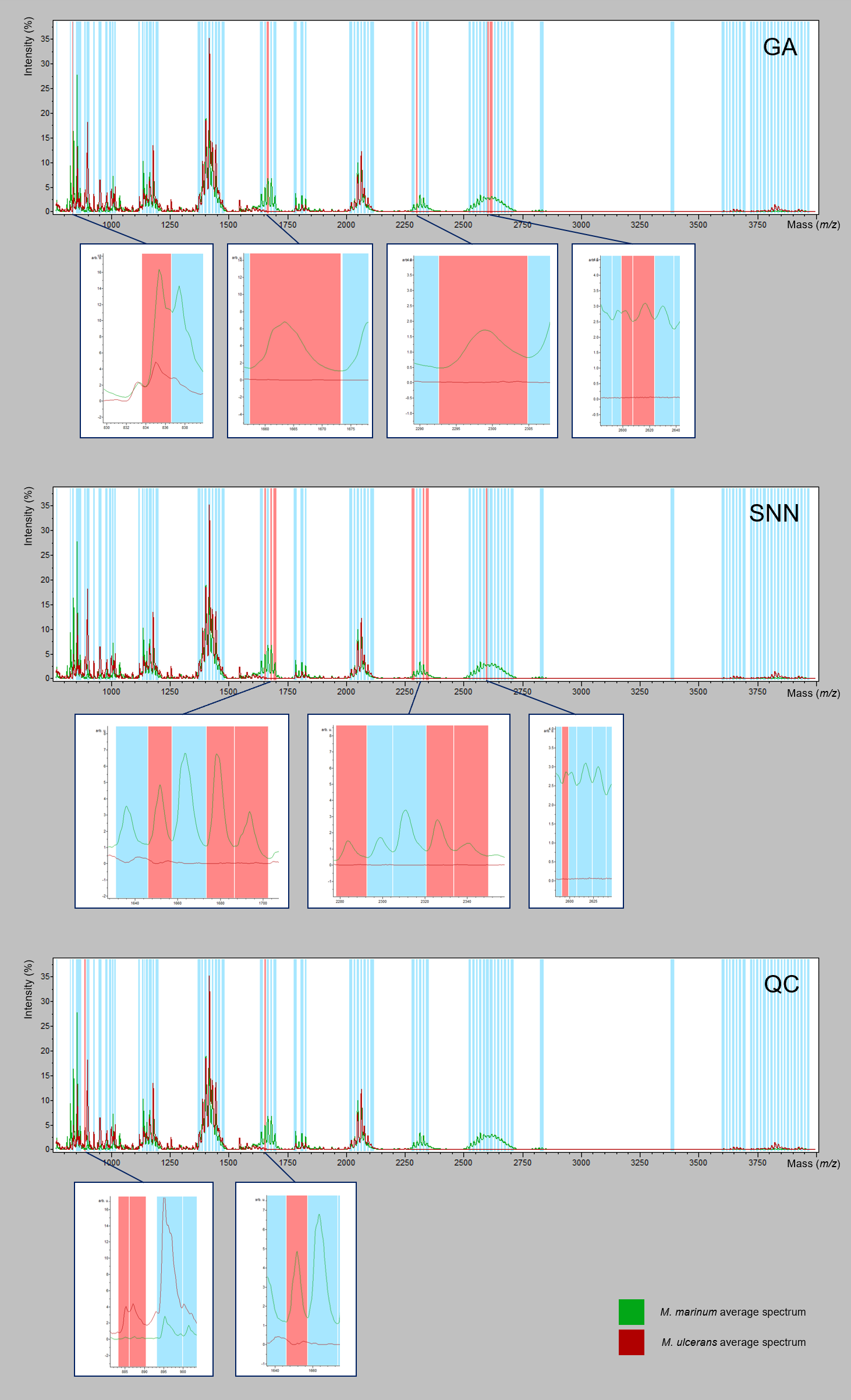
**

**FIG S2**

**
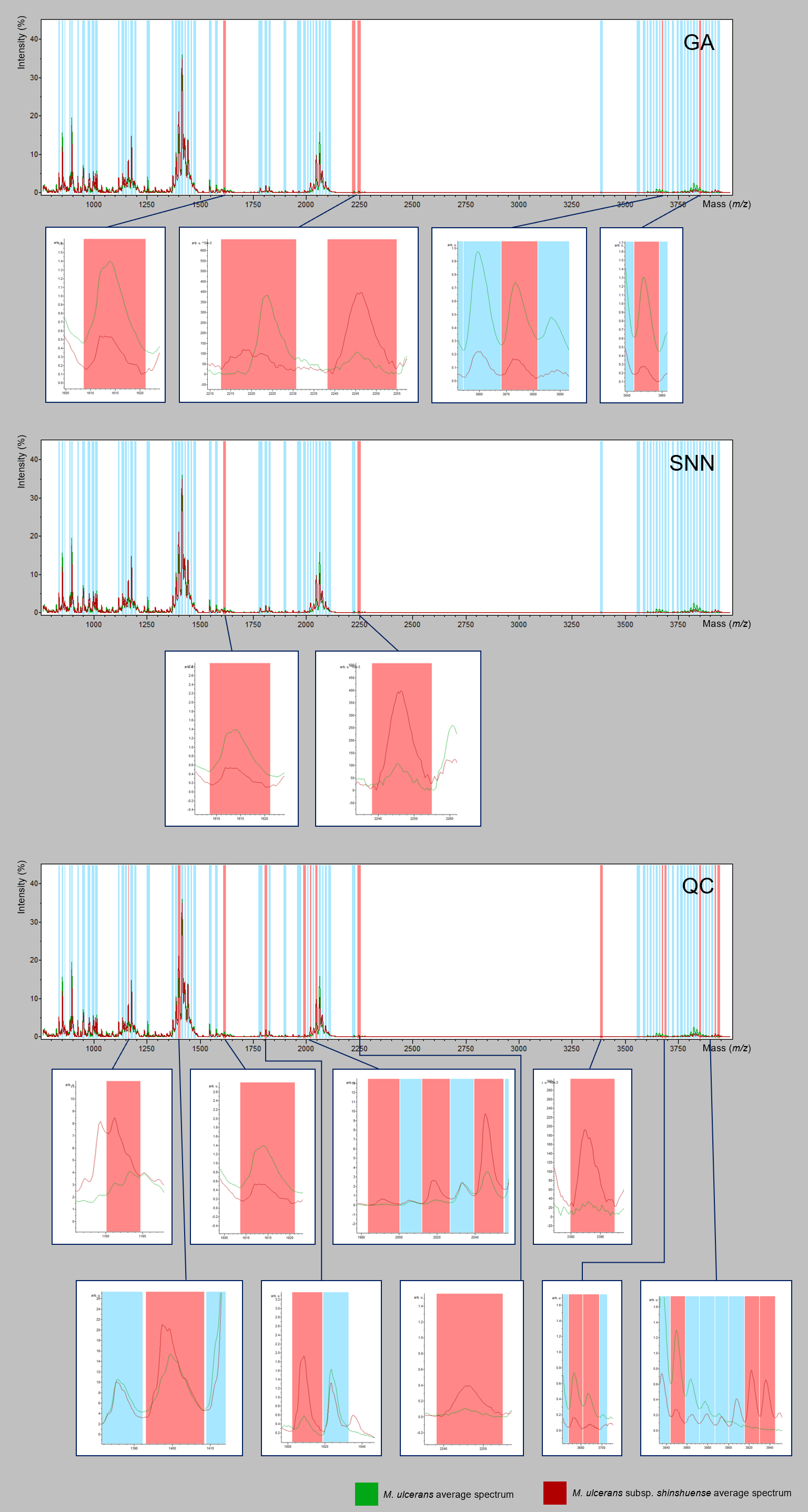
**

**FIG S3**

**
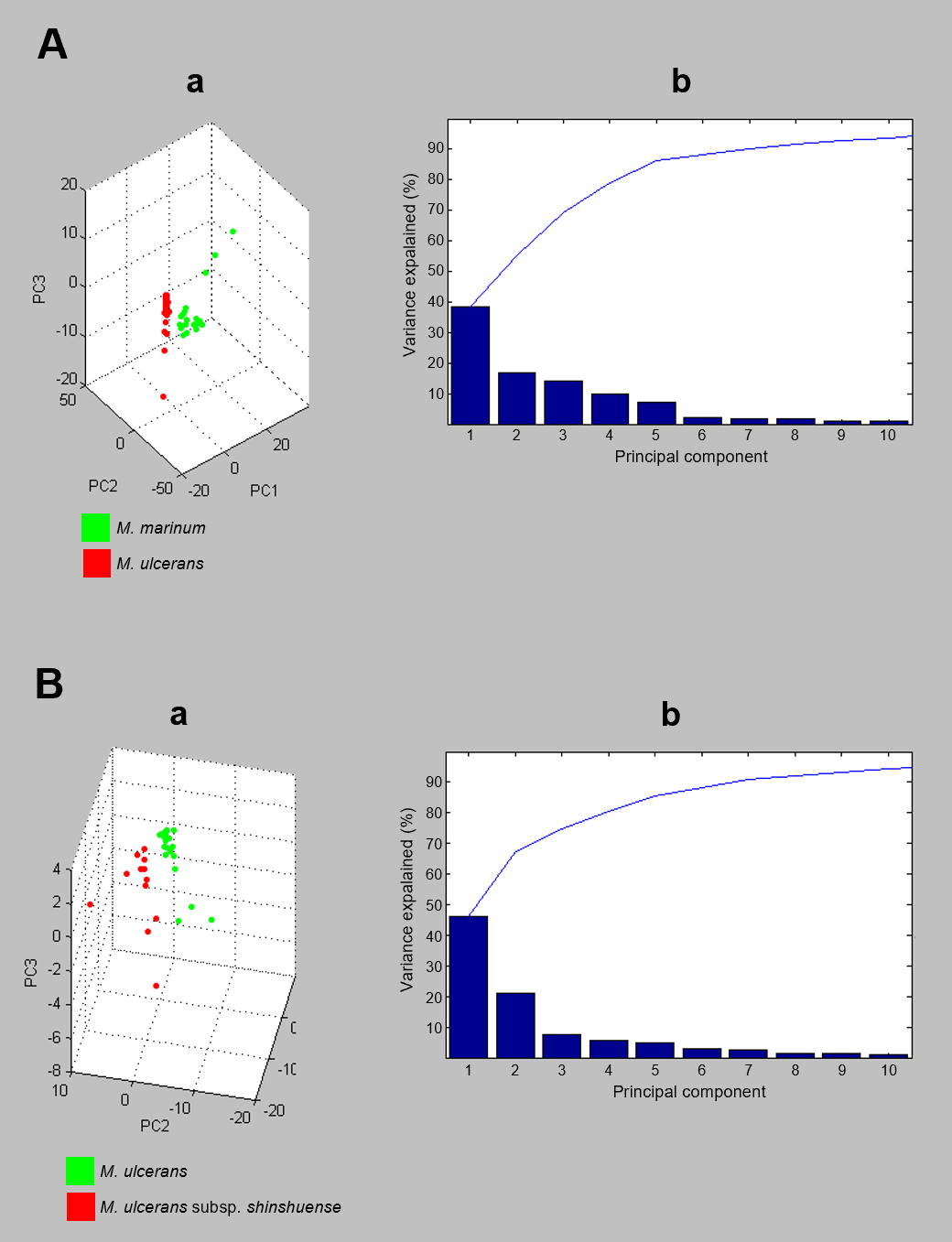
**

**TABLE S1** Mycobacterial strains used for MALDI-TOF MS measurements

| Species | Strain | Geographic location | Year | Proteomic identification^a^ | 16S rRNA gene^b^ | IS*2404*^c^ | *M. marinum*  lineage^d^ |
| --- | --- | --- | --- | --- | --- | --- | --- |
| *Mycobacterium ulcerans* | ITM 94-1326 | Australia | missing | ND | Mu | + | NA |
|  | ITM 94-1327 | Australia | missing | ND | Mu | + | NA |
|  | ITM 97-107 | Benin | missing | ND | Mu | + | NA |
|  | ITM 5147 | Australia | missing | ND | Mu | + | NA |
|  | ITM 94-1324 | Australia | 1994 | ND | Mu | + | NA |
|  | ITM 9550 | Australia | 1983 | ND | Mu | + | NA |
|  | ITM 94-659 | Ivory Coast | missing | ND | Mu | + | NA |
|  | ITM 94-511 | Ivory Coast | 1994 | ND | Mu | + | NA |
|  | ITM 94-1328 | Malaysia | 1994 | NH | Mu | + | NA |
|  | ITM 97-96 | Benin | missing | ND | Mu | + | NA |
|  | ITM 97-680 | Togo | missing | ND | Mu | + | NA |
|  | Agy99 | Ghana | 1999 | Mm | Mu | + | NA |
| *Mycobacterium ulcerans* subsp. *shinshuense* | LRC1202 | Japan | 2012 | ND | Mu_sh | + | NA |
|  | LRC1302 | Japan | 2013 | ND | Mu_sh | + | NA |
|  | LRC1305 | Japan | 2013 | Mm | Mu_sh | + | NA |
|  | LRC1306 | Japan | 2013 | ND | Mu_sh | + | NA |
|  | LRC1307 | Japan | 2013 | ND | Mu_sh | + | NA |
|  | LRC1308 | Japan | 2013 | ND | Mu_sh | + | NA |
|  | LRC1309 | Japan | 2013 | ND | Mu_sh | + | NA |
|  | LRC1402 | Japan | 2014 | ND | Mu_sh | + | NA |
|  | LRC1403 | Japan | 2014 | ND | Mu_sh | + | NA |
|  | LRC1406 | Japan | 2014 | ND | Mu_sh | + | NA |
|  | LRC1501 | Japan | 2015 | Mm | Mu_sh | + | NA |
|  | LRC1601 | Japan | 2016 | ND | Mu_sh | + | NA |
|  | LRC1702 | Japan | 2017 | ND | Mu_sh | + | NA |
|  | LRC1703 | Japan | 2017 | ND | Mu_sh | + | NA |
|  | LRC1706 | Japan | 2017 | ND | Mu_sh | + | NA |
|  | LRC1801 | Japan | 2018 | ND | Mu_sh | + | NA |
|  | LRC1802 | Japan | 2018 | Mm | Mu_sh | + | NA |
|  | LRC1803 | Japan | 2018 | ND | Mu_sh | + | NA |
|  | LRC1902 | Japan | 2019 | ND | Mu_sh | + | NA |
|  | LRC1903 | Japan | 2019 | ND | Mu_sh | + | NA |
|  | LRC2001 | Japan | 2020 | ND | Mu_sh | + | NA |
|  | LRC2002 | Japan | 2020 | ND | Mu_sh | + | NA |
|  | LRC2101 | Japan | 2021 | Mm | Mu_sh | + | NA |
| *Mycobacterium marinum* | LRC1 | Japan | 2007 | Mm | Mm | - | I |
|  | LRC2 | Japan | 2009 | ND | Mm | - | I |
|  | LRC3 | Japan | 2009 | ND | Mm | - | I |
|  | LRC4 | Japan | 2009 | Mm | Mm | - | I |
|  | LRC5 | Japan | 2010 | ND | Mm | - | I |
|  | LRC6 | Japan | 2011 | ND | Mm | - | I |
|  | LRC7 | Japan | 2011 | Mm | Mm | - | I |
|  | LRC8 | Japan | 2014 | ND | Mm | - | II |
|  | LRC9 | Japan | 2014 | ND | Mm | - | I |
|  | LRC10 | Japan | 2015 | Mm | Mm | - | I |
|  | LRC11 | Japan | 2015 | ND | Mm | - | I |
|  | LRC12 | Japan | 2016 | ND | Mm | - | I |
|  | LRC13 | Japan | 2016 | Mm | Mm | - | I |
|  | LRC14 | Japan | 2016 | ND | Mm | - | II |
|  | LRC15 | Japan | 2017 | ND | Mm | - | I |
|  | LRC16 | Japan | 2020 | Mm | Mm | - | I |
|  | LRC17 | Japan | 2021 | ND | Mm | - | I |
|  | LRC18 | Japan | 2021 | ND | Mm | - | II |
|  | LRC19 | Japan | 2022 | Mm | Mm | - | I |

NA, not applicable; ND, no data; NH, no hits; Mu, *Mycobacterium ulcerans*; Mu_sh, *Mycobacterium ulcerans* subsp. *shinshuense*; Mm, *Mycobacterium marinum*.

^a^ Results of identification using a proteomic method, the MALDI Biotyper Sirius system with the MBT Compass HT software v. 5.1.400 (Bruker, Germany).

^b^ Results of identification based on 16S rRNA gene (1).

^c^ Results of the insertion sequence (IS) *2404* detection (2).

^d^ Lineage of *M. marinum* proposed by Das et al. (2018) (3).
